# Exposure to Structural Sexism at Birth is Associated with Faster Late‐Life Memory Decline Among Black and White Women in the U.S.

**DOI:** 10.1002/alz.087091

**Published:** 2025-01-09

**Authors:** Justina F Avila, Paris AJ Adkins‐Jackson, Tanisha G Hill‐Jarrett, Adam M. Brickman, Nicole Schupf, Richard Mayeux, Jennifer J. Manly

**Affiliations:** ^1^ Columbia University Irving Medical Center, New York, NY USA; ^2^ Columbia University, New York, NY USA; ^3^ University of California, San Francisco, San Francisco, CA USA; ^4^ Department of Neurology, Columbia University, New York, NY USA

## Abstract

**Background:**

Structural determinants of health may be important drivers of late‐life cognitive decline and dementia risk for women who have a disproportionate burden of Alzheimer’s disease (AD). This study examines how early life exposure to structural sexism influences cognitive trajectories among women racialized as Black and White.

**Method:**

This study analyzed longitudinal data from two cohort studies: the Washington Heights‐Inwood Columbia Aging Project (WHICAP) and the Health and Retirement Study (HRS). Eligible participants were aged 60+, US born, non‐Latinx, and dementia‐free at their baseline cognitive assessment. Confirmatory factor analysis produced a latent variable of state‐level structural sexism comprised of 6 US Census‐derived indicators representing sex/gender disparities in access to economic, political, cultural, and reproductive health resources. Latent variable scores, per US state, were generated for each year between 1902 ‐ 1954 and linked with WHICAP and HRS participants using birth year and state. Cognitive outcomes were level and change in memory performance. Stratified mixed‐effects models were used to examine associations between structural sexism and memory trajectories across women and men. Interaction terms were included to examine associations by racialized group.

**Result:**

Exposure to higher levels of structural sexism was associated with lower baseline memory performance among WHICAP women and a more rapid rate of memory decline among women in both studies. For women, the difference in rate of memory decline between being born in the state with the highest structural sexism versus the state with the lowest structural sexism was equivalent to 8 to 9 years of cognitive aging. Associations between structural sexism and baseline memory were stronger among Black women compared with White women for both studies.

**Conclusion:**

This study provides some of the first insights into the relationship between structural sexism and late‐life cognitive aging outcomes. Results suggest that, among US‐born Black and White women, early‐life exposure to structural sexism negatively impacts late‐life memory trajectories. Structural determinants are actionable risk factors that can be modified through policy changes. A deeper understanding of the pathways linking structural sexism to AD risk can inform the development of future policy interventions to improve late‐life cognitive health among women.

